# Parents’ and Teachers’ Perceptions of Comprehensive Sexuality Education in Early Childhood and Primary Education in Greece

**DOI:** 10.3390/ejihpe16070095

**Published:** 2026-06-30

**Authors:** Vasiliki Katsarou, Vassiliki Pliogou, Maria Gasouka, Evaggelia Kalerante

**Affiliations:** 1 Department of Early Childhood Education, School of Social Sciences and Humanities, University of Western Macedonia, 53100 Florina, Greece; vpliogou@uowm.gr (V.P.); ekalerante@uowm.gr (E.K.); 2 Department of Preschool Education Science and Educational Design, University of the Aegean, 85131 Rhodes, Greece; mgasouka@aegean.gr

**Keywords:** comprehensive sexuality education, parents, teachers, early childhood education, primary education, perceptions

## Abstract

This study examines parents’ and teachers’ perceptions of Comprehensive Sexuality Education (CSE) in preschool and primary school settings in Greece. Using a quantitative survey design, data were collected from 326 parents and 212 teachers. The study explored perceptions of the importance of CSE, appropriate age and content, implementation barriers, and the influence of socio-demographic and ideological factors. Both groups expressed strong support for CSE, particularly when framed as a means of protecting children and providing reliable information. However, support appeared conditional and selective, with greater hesitation toward topics directly related to sexuality and sexual behaviour. Significant differences emerged, with parents expressing greater concerns regarding age appropriateness and potential negative consequences, while teachers showed stronger support for earlier and more comprehensive implementation. Both groups endorsed a developmental approach, with increasing acceptance of more complex topics at older ages. Although statistically significant differences emerged in several areas, effect sizes were generally small, indicating substantial overlap between parents’ and teachers’ views. Teachers were more likely to identify key implementation barriers, including lack of training, limited resources, and parental resistance. Ideological orientation appeared to be consistently associated with the perceived importance of CSE across both samples.

## 1. Introduction

Sexuality education has increasingly been recognized as an essential component of children’s wellbeing, rights, and social development (Fisher et al., 2015; Morawska et al., 2015). The international community has moved toward a more consensual and holistic understanding of comprehensive sexuality education (CSE), shifting away from earlier approaches that primarily emphasised disease and risk prevention. CSE is now conceptualised as a comprehensive educational process grounded in human rights, gender equality, and respect for diversity, aiming to support children’s overall development and wellbeing (UNFPA et al., 2020). Within this framework, it is also viewed as a means of promoting gender equality and challenging stereotypes from the early years (Singh et al., 2025).

International organisations such as UNESCO (2018) emphasise that CSE constitutes a gradual learning process that can begin in early childhood through age-appropriate and developmentally sensitive approaches. In particular, the International Technical Guidance on Sexuality Education (UNESCO, 2018) proposes a framework organised around eight key concepts, each encompassing age-specific topics ranging from early childhood (5 years) to adulthood (18+). These include: (1) relationships; (2) values, rights, culture and sexuality; (3) understanding gender; (4) violence and staying safe; (5) skills for health and well-being; (6) the human body and development; (7) sexuality and sexual behaviour; and (8) sexual and reproductive health.

However, the implementation of CSE in early childhood settings varies considerably across countries and educational systems (Ketting & Ivanova, 2018; Picken, 2020). Public debates frequently frame CSE as a topic primarily relevant to older students, while early childhood settings are often perceived as spaces where such discussions should be limited mainly to biological or risk-prevention elements (Cacciatore et al., 2020; Picken, 2020; Robinson & Davies, 2017).

In some contexts, it is integrated into broader health or citizenship education programmes, while in others it is addressed informally through teacher responses to children’s questions (Ketting & Ivanova, 2018; Prioletta et al., 2024). At the same time, research indicates that children begin to express curiosity about bodies, gender differences, reproduction, and relationships from an early age. This raises questions about how educational systems should respond to these developmental realities (K. A. Martin & Torres, 2014).

This distinction becomes particularly important in the Greek context, where CSE tends to be fragmented and often limited to specific thematic areas. It is primarily delivered through the Skills Labs on sexuality education, which constitute the main institutional mechanism for introducing relevant content, mainly in primary education (Ministry of Education, 2021). However, their implementation remains limited, as SL are compulsory only in specific grades (Grades 3 and 6), while overall they remain fragmented and largely optional (Katsarou & Pliogou, 2026).

To provide a more integrated understanding of attitudes toward CSE, the present study draws on complementary theoretical perspectives. From a gender perspective, sexuality and gender are understood as socially constructed and shaped through norms and practices, which influence how CSE content is perceived and evaluated (Butler, 1990; Connell, 2009). From a moral perspective, attitudes toward CSE can be interpreted through broader value frameworks that shape judgments about what is appropriate for children. Moral orientations related to protection, authority, and social norms may lead to differentiated acceptance of specific CSE topics (Haidt, 2012). Finally, from a socio-cultural perspective, attitudes are formed through social interaction and are embedded within cultural and institutional contexts (Vygotsky, 1978).

Within this framework, political ideology, religiosity, and educational level were selected as key factors because they have been consistently identified in the literature as important influences on attitudes toward Comprehensive Sexuality Education (CSE). Political ideology and religiosity can be understood as value-based orientations that shape moral evaluations of sexuality, gender, family life, and children’s education. Research suggests that individuals with more conservative political views and higher levels of religiosity tend to express greater reservations regarding specific domains of CSE, particularly those related to gender diversity, sexual orientation, and sexual behaviour (Hendriks et al., 2024; Hurst et al., 2023; Ketting & Ivanova, 2018; Steadman et al., 2014). Educational level has also been associated with attitudes toward CSE, as higher educational attainment is often linked to greater acceptance of diversity, stronger trust in scientific knowledge, and more positive attitudes toward evidence-based educational practices, although findings remain less consistent across studies. While other factors, such as age, gender, professional experience, cultural background, parental status, and previous exposure to sexuality education, may also influence attitudes toward CSE, these three variables were selected because they represent broader socio-cultural and value-based dimensions that have been repeatedly highlighted in international research and align closely with the theoretical framework adopted in the present study.

## 2. Literature Review

### 2.1. Parents’ Views on CSE

Research consistently identifies parents as central actors in children’s early sexual socialisation. Children’s understanding of the body, relationships, and social norms is shaped through interaction with significant adults, particularly parents, who play a key role in shaping children’s perceptions (Vygotsky, 1978). Early communication about the body, relationships, and safety can promote healthy attitudes, strengthen protective behaviours, and support children’s emotional development (Morawska et al., 2015). However, studies show that many parents experience discomfort, lack of confidence, or uncertainty when discussing sexuality with their children, particularly at younger ages (Morawska et al., 2015; Shin et al., 2019).

Parents’ perceptions toward CSE depend strongly on its content and the age at which it is introduced. Topics such as body knowledge, hygiene, and abuse prevention are widely accepted in primary education, while themes related to reproduction, gender diversity, or sexual health tend to generate greater hesitation when introduced at earlier ages (Depauli & Plaute, 2018). This pattern can be interpreted through the dominant “innocent child” discourse, which constructs children as asexual, vulnerable, and in need of protection, thereby legitimising the inclusion of protective topics while problematising the introduction of sexuality-related content at early ages (Robinson, 2008).

At the same time, concerns about children’s exposure to digital media and online sexual content have increased parental support for school-based sexuality education as a source of reliable information (Moran & Van Leent, 2022; Robinson et al., 2017). Research also highlights that parents often perceive sexuality education as a shared responsibility between family and school. While some emphasise the primary role of the family, many support the involvement of schools provided that teaching is age-appropriate, transparent, and delivered by well-trained professionals (Robinson & Davies, 2017).

Religious ideologies play a significant role in shaping parental attitudes, influencing both the values transmitted within the family and perceptions of what is appropriate for children to learn (Dent & Maloney, 2017; Ketting & Ivanova, 2018). Empirical evidence indicates that higher levels of religious conservatism and more frequent engagement in religious practices are associated with lower levels of support for CSE (Steadman et al., 2014). However, this relationship is not uniform, as religiosity does not necessarily lead to a general rejection of CSE but appears to influence the acceptance of specific content areas, particularly those related to gender, sexual orientation, and relationships (Ketting & Ivanova, 2018).

At the same time, political orientation interacts with religiosity in shaping attitudes toward CSE. Studies indicate that more liberal or centre-left parents tend to express higher levels of support for CSE, whereas more conservative orientations are associated with more restrictive views, particularly regarding sensitive topics (Hendriks et al., 2024). Political and religious beliefs appear to influence the content and model of sexuality education rather than its overall acceptance (Steadman et al., 2014; Kantor & Levitz, 2017). Moreover, lower support for specific topics—such as sexual identity, pleasure, and practical skills—is most pronounced when high religiosity coexists with political conservatism, whereas in the absence of this combination, parents tend to endorse a broader range of CSE themes (Hurst et al., 2023).

### 2.2. Teachers’ Preparedness for CSE

Research demonstrates that educators in early childhood settings often conceptualise CSE primarily in terms of body knowledge, personal hygiene, and safety (Prioletta & Philibert, 2025). Nevertheless, concerns about age appropriateness and parental reactions remain common, with teachers sometimes avoiding certain topics due to fear of criticism or uncertainty about institutional expectations (Cumming-Potvin & Martino, 2018).

Teachers play a central role in the implementation of CSE, as they translate policy frameworks into classroom practice and shape how sexuality-related knowledge is introduced in the early years of schooling. Research consistently shows that teachers generally support the inclusion of CSE and recognise its importance for children’s development, health, and wellbeing (Prioletta et al., 2024).

However teachers’ conceptions of CSE vary according to educational level. In early childhood settings, CSE is typically framed as body awareness and self-protection. Preschool teachers emphasise helping children recognise body parts, understand privacy, and identify appropriate and inappropriate touch often delivered through play-based activities, storytelling, and everyday “teachable moments” (Prioletta et al., 2024; Rakhmawati et al., 2022). In primary education, teachers tend to link CSE to emotional development, relationships, and responsible decision-making (Depauli & Plaute, 2018; Plaza-del-Pino et al., 2021; Zhuravleva & Helmer, 2024).

Despite this generally positive attitude, teachers’ preparedness is shaped by multiple professional and contextual factors. Some educators emphasise the need for expert-professionals or trained teachers, while others highlight the importance of communication skills, confidence, and comfort in discussing sensitive topics (Burns & Hendriks, 2018; Zhuravleva & Helmer, 2024). Teachers also stress the importance of cooperation between schools and families, noting that parental support increases their confidence and facilitates implementation (Yalkilic & Yağan, 2023). Professional development, institutional support, and access to teaching materials are consistently identified as key facilitators (Depauli & Plaute, 2018).

At the same time, research highlights several barriers that limit teachers’ readiness to implement CSE. The most frequently cited challenge is the lack of adequate training, with many teachers reporting limited preparation and uncertainty in responding to children’s questions or designing age-appropriate activities (Hendriks et al., 2024; Shibuya et al., 2023). Parental reactions constitute another major concern, as teachers often fear complaints or misunderstandings when sexuality-related topics are discussed (Ullman et al., 2022). Cultural norms and social taboos may further increase teachers’ hesitation, particularly regarding gender and sexuality diversity (Ezer et al., 2020; Zulu et al., 2019). In addition, structural constraints—such as limited time, inadequate resources, and insufficient institutional support—further restrict implementation (Depauli & Plaute, 2018; P. Martin et al., 2020; Plaza-del-Pino et al., 2021; Shibuya et al., 2023).

Finally, teachers’ perceptions and practices are influenced by broader demographic and contextual factors. Research indicates that younger and more highly educated teachers tend to be more receptive to holistic and inclusive approaches, whereas older teachers or those from more conservative backgrounds often express greater hesitation (Shibuya et al., 2023). Gender differences are also evident, with female teachers placing greater emphasis on care and protection and male teachers focusing more on biological aspects (Zhuravleva & Helmer, 2024). More broadly, social and cultural contexts can function either as facilitators or constraints in the integration of CSE, shaping both content and pedagogical approaches (Rivenes Lafontan et al., 2024; Chavula et al., 2022). However, most existing research focuses on adolescence, with limited evidence available for early childhood education.

### 2.3. The Current Study

Although international research has examined parental and teacher perceptions on CSE, important gaps remain. First, the literature primarily focuses on adolescents or secondary education (Noorman et al., 2022), with early childhood and primary school contexts comparatively under-researched. Second, parents and teachers are typically examined separately, despite the fact that effective CSE depends on their interaction. Moreover, in the Greek context, empirical evidence is particularly limited. Addressing these gaps, the present study aims to answer the following questions:

1. What are parents’ and teachers’ perceptions of sexuality education in preschool and primary education?
2. What is the appropriate age for introducing sexuality education, and what content should it include?
3. What factors act as barriers or facilitators in its implementation?
4. How do socio-demographic and ideological factors influence the perceived importance of CSE among parents and teachers?

## 3. Materials and Methods

### 3.1. Study Design

The present study employed a quantitative, survey-based design to examine parents’ and teachers’ perceptions and views regarding the implementation of CSE in school contexts. This approach enabled the collection of structured data from a large sample and the use of statistical analyses to explore group differences, levels of acceptance, and factors influencing the implementation of CSE.

The study relied on non-probability convenience sampling, as data were collected through online distribution. While this approach facilitated broad participation, it may have introduced sampling bias, including the overrepresentation of certain groups (e.g., female participants). Therefore, the findings should be interpreted as indicative rather than fully generalizable to the broader population.

### 3.2. Participants and Setting

The study was conducted in the Region of Epirus, Greece. The target population included teachers working in kindergarten and primary education, as well as parents of children enrolled in these settings. The final sample consisted of 326 parents and 212 teachers.

The sample was heterogeneous in terms of demographic characteristics. All participating parents had at least one child attending kindergarten or primary school. Female participants were overrepresented in both groups. While this distribution may not fully reflect the parent population, this pattern is consistent with the gender distribution typically observed in early childhood and primary education settings in Greece.

### 3.3. Procedure

The study was approved by the Research Ethics Committee of the University of Western Macedonia (Protocol No. 146/2025). Quantitative data were collected through online questionnaires administered via Google Forms from April to September 2025. This platform was selected due to its accessibility, automated data recording, and ability to ensure participants’ anonymity. The online distribution also enabled participation from different geographical areas within the Epirus region.

The questionnaires were initially disseminated through institutional school networks. For teachers, the survey links were distributed via school email accounts, while for parents distribution was facilitated through the schools attended by their children. Participation was entirely voluntary, and no incentives were provided.

Prior to completing the questionnaire, participants were informed about the purpose of the study, anonymity, and their right to withdraw. The information sheet stated that by proceeding to complete the questionnaire, participants indicated their consent to participate, in line with ethical principles for research in the social sciences.

### 3.4. Measures

Two separate questionnaires were developed for parents and teachers, including both common and group-specific sections. Because the aim of the study was to examine multiple dimensions of perceptions toward CSE, including its perceived importance, age appropriateness, content, implementation barriers, preferred providers, and educational policy, no single validated instrument was identified that adequately covered all of these domains for both parents and teachers in the Greek preschool and primary education context. Therefore, the questionnaires were developed by adapting items from multiple previously validated instruments and international frameworks. This approach enabled the inclusion of conceptually distinct aspects of CSE that have been highlighted in the literature as important components of parents’ and teachers’ perceptions.

The instruments were constructed based on items adapted from multiple validated questionnaires used in previous research on sexuality education (Ballal et al., 2022; Ezer et al., 2020; Fisher et al., 2015; Hendriks et al., 2024; Hurst et al., 2023; Robinson & Davies, 2017; Shin et al., 2019; Ullman et al., 2022; UNESCO, 2018; Wood et al., 2022; Zhang & Yuan, 2023). Several items were identified as common across more than one instrument, supporting their inclusion and strengthening content validity.

For the purposes of this study, only the sections common to both questionnaires were included in the analysis: (a) demographic characteristics, (b) perceptions of CSE, (c) views on its provision within the school context and (d) perceived barriers to implementation. Only the sections relevant to the aims of the present study are reported here.

The questionnaires included a range of item formats, including five-point Likert-scale items (1 = not at all to 5 = very much), single-choice items, and multiple-response items.

The perceived importance of CSE was assessed using a single-item measure on a five-point Likert scale.

Religiosity and political orientation were assessed using single-item measures adapted from previous research (Hurst et al., 2023). Religiosity was operationalized and measured exclusively as frequency of religious attendance, assessed with a single-item measure (1 = less than once a year or not at all to 5 = more than once a week), following previous research (Hurst et al., 2023; Koenig & Büssing, 2010). Political orientation was assessed through self-placement on an ideological continuum (1 = very conservative to 5 = very liberal), a widely used approach in social research (Carney et al., 2008).

Items assessing the eight key concepts of CSE and the proposed age groups (5–6, 7–9, 9–12) were based on the International Technical Guidance on Sexuality Education (UNESCO, 2018).

A pilot study was conducted prior to the main data collection to assess the clarity, comprehensibility, and content validity of the questionnaire, as well as the functionality of the online format. The pilot sample included four independent researchers with expertise in quantitative methods and questionnaire design, as well as 12 teachers. Based on their feedback, minor revisions were made to improve item wording, structure, and clarity.

Given the multidimensional nature of the questionnaire and the fact that the items addressed conceptually distinct domains (e.g., perceived importance, age appropriateness, educational content, implementation barriers, preferred providers, and policy perceptions), the construction of composite scales was not considered theoretically appropriate. Consequently, each item was analysed independently and interpreted as reflecting a specific aspect of participants’ perceptions rather than a unified attitudinal construct. Several key variables, including perceived importance of CSE, political self-identification, and religious attendance, were assessed using single-item measures because they referred to conceptually concrete and easily understood constructs. Political self-identification and religious attendance were measured using approaches commonly employed in previous research on attitudes toward CSE and related social issues (Carney et al., 2008; Hurst et al., 2023; Koenig & Büssing, 2010).

### 3.5. Data Analysis

The quantitative data were analysed using IBM SPSS Statistics, Version 31.0 (IBM Corp., Armonk, NY, USA). Descriptive statistics, including frequencies, percentages, means, and standard deviations, were calculated to summarise demographic characteristics and key study variables.

Group differences between parents and teachers were examined using chi-square (χ^2^) tests for categorical variables. For ordinal variables, including Likert-scale responses, the Mann–Whitney U test was employed. Associations between ordinal variables were assessed using Spearman’s rank-order correlation where appropriate.

To examine differences in multiple-response items (e.g., perceived barriers and reasons for or against CSE), chi-square tests were conducted for each item separately. Differences across more than two independent groups (e.g., ideological categories) were examined using the Kruskal–Wallis test.

To examine the combined effect of multiple predictors on the perceived importance of CSE, an ordinal logistic regression analysis was conducted. In the teacher sample, the test of parallel lines indicated that the proportional odds assumption was not violated (*p* = 0.191). However, for the parent sample, the assumption was violated (*p* = 0.017), suggesting that the relationship between predictors and the outcome may vary across response categories. The dependent variable was the perceived importance of CSE, treated as an ordinal outcome variable. For the parent sample, independent variables included educational level, ideological orientation, religious attendance, place of residence, and family income. For the teacher sample, predictors included years of teaching experience, ideological orientation, educational level and religious attendance. Given the violation of the proportional odds assumption in the parent sample, findings from this model should be interpreted with caution. No correction for multiple comparisons was applied.

## 4. Results

### 4.1. Demographic Characteristics

Among parents, the majority were women (80.6%, *N* = 324), as was also the case for teachers (78.2%, *N* = 211). Most parents were aged 40–49 (54.9%, *N* = 326), whereas the largest proportion of teachers were aged 50 years and above (52.8%, *N* = 212) (Table 1).

Regarding educational level, most parents held a university degree (45.1%, *N* = 326), while teachers were more likely to hold a postgraduate degree (51.4%, *N* = 212). In both groups, the majority of participants self-identified as politically centrist (parents: 52.8%; teachers: 44.3%).

Additional characteristics varied by group. Among parents, most identified as Orthodox Christians (87.4%, *N* = 325), reported an annual family income between €10,051 and €20,000 (40.2%, *N* = 323), and resided in urban areas (71.5%). Regarding religious attendance, 203 parents (62.3%) reported attending church “a few times per year”.

Most of the teachers had more than 25 years of teaching experience (35.5%, *N* = 211). Participants were almost evenly distributed between kindergarten (52.9%) and primary school (47.1%, *N* = 210). Regarding religious attendance, the majority reported attending church “a few times per year” (59.2%, *N* = 211).

Due to missing values, the number of valid responses varies across variables.

### 4.2. Perceptions of CSE in Preschool and Primary Education

#### 4.2.1. Perceived Importance of CSE

In both groups, the majority of parents (*Ν* = 288, 88.9%) and teachers (*N* = 205, 96.8%) rated CSE as highly important. Teachers reported a slightly higher mean perceived importance score (M = 4.13, SD = 0.80) compared to parents (M = 3.91, SD = 1.08). A Mann–Whitney U test indicated that there was no statistically significant difference between parents and teachers regarding the perceived importance of CSE in preschool/school-aged children, U = 31,822, *p* = 0.121, r = 0.06.

#### 4.2.2. Integration of CSE into the School Curriculum

To compare parents’ and teachers’ views regarding the integration of CSE into the school curriculum, a chi-square test of independence was conducted. Responses were recoded into two categories for analytical purposes: positive attitude (moderate to high levels of agreement) and other responses (low or no agreement).

The results revealed a statistically significant difference between the two groups, χ^2^(1, *N* = 520) = 6.35, *p* = 0.012. Specifically, high levels of support were observed in both groups, with teachers demonstrating an even higher level of acceptance (91.4%) compared to parents (83.4%). The effect size was small, as indicated by Cramer’s V = 0.11.

#### 4.2.3. Reasons for Providing CSE in Schools

A total of 311 parents and 212 teachers responded to the question regarding the reasons for providing CSE in schools. Participants were allowed to select multiple responses.

As shown in Table 2, both parents and teachers reported high levels of agreement across a range of reasons supporting the provision of CSE. The most frequently endorsed reasons in both groups were “the prevention of sexual abuse” (parents: 85.9%; teachers: 89.2%) and “helping children understand the difference between appropriate and inappropriate touch” (parents: 86.5%; teachers: 88.2%). High proportions of participants also indicated that CSE is necessary “to prevent misinformation from unreliable sources” (parents: 82.0%; teachers: 83.0%).

Additionally, a substantial proportion of respondents highlighted its role in “reducing sexually transmitted infections” (parents: 72.7%; teachers: 59.0%) and “teenage pregnancies” (parents: 72.3%; teachers: 65.6%). Overall, both groups reported high levels of endorsement across most reasons, with only minor variations between them.

In contrast, the least frequently selected reason in both groups was that CSE “prevents children from asking uncomfortable questions” (parents: 17.0%; teachers: 18.9%), suggesting that participants primarily value CSE for its protective and informational role rather than for managing children’s curiosity.

Additional chi-square tests showed a high level of agreement in both groups across most reasons. A statistically significant difference was observed only for the item “reducing sexually transmitted infections”, which was more frequently selected by parents, χ^2^(1, *N* = 523) = 10.73, *p* = 0.001, although the effect size was small (Cramer’s V = 0.14).

#### 4.2.4. Reasons for Not Providing CSE in Schools

Table 3 presents all reported reasons for opposing the provision of CSE in schools among participants who expressed reservations regarding its implementation (parents: *n* = 80; teachers: *n* = 101; total *N* = 181). Because participants were allowed to provide multiple responses and several response options were unique to each group, separate chi-square analyses were conducted only for the response categories shared by both parents and teachers. Parents were significantly more likely than teachers to report that children are “too young” for CSE, χ^2^(1, *N* = 181) = 7.56, *p* = 0.006, Cramer’s V = 0.20; that CSE leads children to “lose their innocence”, χ^2^(1, *N* = 181) = 9.08, *p* = 0.003, Cramer’s V = 0.22; and that it “leads to risky experimentation”, χ^2^(1, *N* = 181) = 6.30, *p* = 0.012, Cramer’s V = 0.19. Effect sizes were small to moderate, indicating modest but meaningful differences between parents and teachers.

#### 4.2.5. Preferred Providers of CSE

A statistically significant difference was found between the two groups (χ^2^(2) = 34.17, *p* < 0.001), with a small to moderate effect size (Cramer’s V = 0.26).

Both parents (50.2%) and teachers (61.3%) most frequently identified health professionals or CSE specialists as the most appropriate providers. However, parents were more likely to consider themselves as suitable providers (25.8%) compared to teachers (9.9%), whereas teachers were more likely than parents to consider themselves as appropriate providers (26.4%).

#### 4.2.6. Preferred Decision-Makers for CSE Content

As participants were allowed to select multiple responses, separate chi-square analyses were conducted for each response category. Statistically significant differences emerged regarding the role of parents, teachers, and the Ministry of Education in decision-making about CSE content. Parents were significantly more likely than teachers to support parental involvement in final decision-making, χ^2^(1, *N* = 537) = 40.33, *p* < 0.001, Cramer’s V = 0.27, indicating a moderate effect size. Smaller but significant differences were also observed for teachers, χ^2^(1, *N* = 537) = 6.68, *p* = 0.010, Cramer’s V = 0.11, and the Ministry of Education, χ^2^(1, *N* = 537) = 5.54, *p* = 0.019, Cramer’s V = 0.10. No differences emerged for other stakeholders.

#### 4.2.7. Characteristics of Teachers Delivering CSE

Both groups overwhelmingly identified specialized training as the most important characteristic for educators providing CSE (parents *N* = 318, 99.1%; teachers *N* = 205, 99.0%), with almost universal agreement in both groups. A statistically significant difference emerged only for the characteristic “friendly personality”, which was more frequently endorsed by teachers than parents, χ^2^(1, *N* = 528) = 4.71, *p* = 0.030, although the effect size was small (Cramer’s V = 0.09). Relatively small proportions of both parents and teachers expressed a preference for same-gender educators in the delivery of CSE. Specifically, (*n* = 30) 9.3% of parents and (*n* = 13) 6.3% of teachers indicated that boys should be taught by male educators and girls by female educators. A small number of isolated responses provided additional insights into participants’ views regarding the characteristics educators should possess in order to provide CSE. Among teachers, individual responses emphasized the importance of overcoming fear of parental reactions and being open-minded, non-judgmental, and inclusive. Among parents, isolated responses referred to the importance of trust-based relationships with students, the belief that educators providing CSE should also be parents themselves, the need for psychiatric certification and absence of religious influence, the preference for younger educators, and the opinion that educators providing CSE should not belong to the LGBTQ+ community. Given their extremely low frequency, these responses cannot be considered representative of the broader sample; however, they may reflect minority positions that occasionally emerge in public debates surrounding comprehensive sexuality education.

### 4.3. Appropriate Age and Content of CSE

#### 4.3.1. Appropriate Age for the Introduction of CSE

A statistically significant difference was observed between parents and teaches (χ^2^(5) = 62.11, *p* < 0.001). The effect size was moderate (Cramer’s V = 0.34), indicating a divergence in the perceptions of the two groups.

Specifically, the majority of parents (51.4%) considered primary school (ages 7–12) as the most appropriate stage for the introduction of CSE, while 29.2% supported its introduction during preschool years (ages 4–6), and a smaller proportion (10.8%) preferred its introduction at lower secondary level (10.8%).

In contrast, the majority of teachers (68.8%) supported introducing CSE during early childhood (ages 1–6), whereas 28.7% considered primary school (ages 7–12) as the appropriate stage.

#### 4.3.2. Perceived Importance of CSE Key Concepts

The aggregated results (Table 4) show that both groups attributed importance to a broad range of CSE concepts. Both groups converged in assigning the lowest priority to “Sexuality and sexual behaviour” (parents: 7.5%; teachers: 8.5%).

However, some differences emerged in the prioritisation of key concepts. Parents placed greater emphasis on “Human body and development” (17.5%), whereas teachers prioritised “Skills for health and wellbeing” (15.6%). Additionally, teachers reported slightly higher percentages for concepts related to values and gender, such as “Values, rights, culture and sexuality” and “Understanding gender”.

Overall, while both groups recognised the multidimensional nature of CSE, teachers appeared to reflect a relatively broader perspective compared to parents.

#### 4.3.3. Age Appropriateness of CSE Key Concepts

In response to the question “At which age level should each topic be taught?” parents and teachers were asked to select among the following age categories: “5–6 years”, “7–9 years”, “9–12 years”, or “should not be taught” (Table 5).

The comparative analysis across age groups reveals a clear developmental progression in both parents’ and teachers’ responses. At ages 5–6, relational and protective topics were prioritised, with “Relationships” emerging as the most prominent concept (parents: 5.74%; teachers: 5.77%), followed by “Skills for health and well-being” (parents: 3.06%; teachers: 4.19%). In the 7–9 age group, emphasis shifted toward “Skills for health and well-being” (parents: 5.91%; teachers: 6.08%) followed by “Relationships” (parents: 4.28%; teachers: 4.18%). In pre-adolescence (9–12 years), “Skills for health and well-being” (parents: 8.53%; teachers: 8.11%) continued to be the most frequently endorsed concept, and a further shift was observed toward more complex and biologically oriented topics, particularly “Human body and development” (parents: 8.00%; teachers: 7.40%) and “Sexual and reproductive health” (parents: 7.23%; teachers: 6.33%).

At the same time, “Sexuality and sexual behaviour” showed a gradual increase across age groups in both samples (parents: 0.23% → 1.15% → 4.38%; teachers: 0.39% → 0.95% → 3.99%), indicating a progressive acceptance of more complex content as children grow older.

In addition, the total number of responses increased substantially across age groups (parents: 5–6: *N* = 1617; 7–9: *N* = 2448; 9–12: *N* = 4203; teachers: 5–6: *N* = 1407; 7–9: *N* = 1581; 9–12: *N* = 2909), indicating that both parents and teachers adopt a more restrictive approach in early childhood, while progressively endorsing a broader range of topics in later developmental stages.

At the same time, responses indicating that certain topics “should not be taught” remained relatively limited, although higher levels of resistance were observed for more sensitive domains, particularly “Sexual and reproductive health” (parents: 1.34%; teachers: 1.70%).

Overall, both groups demonstrate a similar developmental logic, with an expanding range of acceptable topics across age groups and a gradual shift from social and relational themes toward biological and reproductive aspects of CSE.

### 4.4. Barriers and Facilitators in the Implementation of CSE

#### 4.4.1. Barriers to Implementation of CSE

Table 6 presents parents’ and teachers’ perceptions regarding barriers to the implementation of CSE in primary education. Teachers more frequently identified structural and social barriers, including lack of training [χ^2^(1, *N* = 533) = 15.49, *p* < 0.001, Cramer’s V = 0.17], parental fear [χ^2^(1, *N* = 533) = 17.95, *p* < 0.001 Cramer’s V = 0.18], societal conservatism [χ^2^(1, *N* = 533) = 6.65, *p* = 0.010 Cramer’s V = 0.11], and parental resistance to the implementation of CSE [χ^2^(1, *N* = 533) = 6.77, *p* = 0.009 Cramer’s V = 0.11]. The largest difference concerned teachers’ discomfort in teaching CSE, reported by 65.1% of teachers compared to 32.4% of parents, χ^2^(1, *N* = 533) = 53.75, *p* < 0.001, Cramer’s V = 0.32, indicating a moderate effect size. Smaller but significant differences were also observed for lack of training, parental fear, societal conservatism, and parental resistance, although effect sizes remained small. Among these was a single parent’s response identifying references to LGBTQI+ issues as a potential barrier to the provision of sexuality education.

#### 4.4.2. Handling Children’s Questions

Both groups supported the view that teachers should use children’s questions about sexuality as opportunities for discussion. This position was more strongly endorsed by teachers (92.4%) than parents (82.4%). In contrast, parents were more likely than teachers to believe that children should be referred to their parents for such discussions. Overall, the distribution of responses differed significantly between the two groups, χ^2^(4, *N* = 530) = 11.77, *p* = 0.019, although the effect size was small (Cramer’s V = 0.15). Very small proportions in both groups supported avoiding discussion altogether or believed that sexuality-related topics should not be discussed in schools. A small number of participants in both groups also indicated that such discussions should take place only under specific conditions, such as appropriate teacher training or cooperation with parents.

#### 4.4.3. LGBTQ+ Topics

Responses reflecting negative attitudes toward discussing these topics were reported at very low levels in both groups. The dominant view in both groups was that schools should provide neutral, informative explanations without value judgment. Most parents and teachers supported the view that schools should provide factual information about homosexuality and LGBTQI+ issues without explicitly framing homosexuality as either wrong or acceptable. This position was endorsed by 65.4% of parents and 62.7% of teachers. At the same time, teachers were more likely than parents to support teaching that homosexuality is acceptable (33.5% vs. 26.1%), whereas parents were more likely to endorse restrictive responses, including the view that schools should not discuss homosexuality at all (5.0% vs. 3.3%) or that homosexuality should be presented as wrong (3.5% vs. 0.5%). Overall, the distribution of responses differed significantly between the two groups, χ^2^(3, *N* = 527) = 8.26, *p* = 0.041, although the effect size was small (Cramer’s V = 0.13).

#### 4.4.4. Perceived Societal Readiness

Parents were more likely than teachers to perceive Greek society as unprepared to accept CSE in schools (44.9% vs. 34.3%), whereas teachers expressed more moderate positions, including slightly higher levels of neutrality and disagreement. The distribution of responses differed significantly between the two groups, χ^2^(2, *N* = 533) = 6.02, *p* = 0.049; however, the effect size was small (Cramer’s V = 0.11), indicating relatively limited differences overall.

#### 4.4.5. Evaluation of Educational Policy

Both groups evaluated the current educational policy regarding CSE as largely inadequate. Parents expressed more negative views than teachers, with 94.7% considering current policy either “not at all adequate” or “slightly adequate,” compared to 87.6% of teachers. The distribution of responses differed significantly between the two groups, χ^2^(1, *N* = 529) = 6.61, *p* = 0.010, although the effect size was small (Cramer’s V = 0.10).

### 4.5. Factors Influencing the Perceived Importance of CSE

#### 4.5.1. Political Self-Identification

To examine the relationship between political self-identification and perceived importance toward CSE, the Kruskal–Wallis test was applied.

In the parent sample, statistically significant differences were observed across ideological groups (H(3) = 32.06, *p* < 0.001). Parents who identified as “very liberal” reported higher levels of perceived importance of CSE (M = 4.42, SD = 1.00), followed by those who were “somewhat liberal” (M = 4.29, SD = 0.77) and “ centrist” (M = 3.76, SD = 1.05), whereas “somewhat conservative” parents reported lower mean scores (M = 3.47, SD = 1.23). The effect size indicated a moderate association between political ideology and perceived importance of CSE (η^2^ = 0.10).

A similar pattern was observed in the teacher sample, where statistically significant differences were also found (H(3) = 11.45, *p* = 0.009). Teachers who identified as “very liberal” reported higher levels of perceived importance of CSE (M = 4.59, SD = 0.50), whereas “somewhat conservative” teachers reported lower levels of perceived importance (M = 4.00, SD = 0.57) (Table 7). The effect size was smaller in the teacher sample (η^2^ = 0.056), suggesting a weaker but still meaningful association between political ideology and perceived importance of CSE.

Overall, these findings suggest that political ideology is associated with perceptions of the importance of CSE, particularly among parents, where ideological differences appeared more pronounced.

#### 4.5.2. Religious Attendance

The relationship between religious attendance (measured on a six-point ordinal scale) and perceived importance of CSE for the parents sample was examined using Spearman’s rank-order correlation. A statistically significant negative correlation was found (ρ = −0.230, *p* < 0.001, *N* = 326), indicating that higher levels of religious attendance were associated with lower evaluations of the importance of CSE.

For the teacher sample, the relationship between religious attendance and perceived importance of CSE was also examined using Spearman’s rank-order correlation. A statistically significant negative correlation was found (ρ = −0.173, *p* = 0.012, *N* = 209), indicating that higher levels of religious attendance were associated with lower evaluations of the importance of CSE, although the effect size was small.

#### 4.5.3. Multivariate Analysis (Parents)

To examine the combined effect of multiple factors on the perceived importance of CSE, an ordinal logistic regression analysis was conducted. The dependent variable was the perceived importance of CSE. The independent variables included parental educational level, household income, place of residence, political self-identification, religious attendance.

For the parent sample, the ordinal logistic regression model was statistically significant (χ^2^(6) = 55.20, *p* < 0.001), with a Nagelkerke R^2^ = 0.18. Political self-identification (β = 0.679, *p* < 0.001), educational level (β = 0.504, *p* < 0.001), and frequency of religious attendance (β = −0.360, *p* = 0.012) were statistically significant predictors within the model of the perceived importance of CSE, although the overall explanatory power of the model was modest. Specifically, more liberal participants and those with higher educational levels tended to report higher perceived importance, whereas higher levels of religious attendance were associated with lower perceived importance.

In contrast, household income (β = −0.220, *p* = 0.071) and place of residence (β = −0.671, *p* = 0.127) were not statistically significant predictors (Table 8).

#### 4.5.4. Multivariate Analysis (Teachers)

To examine the combined effect of multiple factors on the perceived importance of CSE, an ordinal logistic regression analysis was conducted. The dependent variable was the perceived importance of CSE. The independent variables included years of teaching experience, educational level, political self-identification, and religious attendance.

For the teacher sample, the model was statistically significant (χ^2^(4) = 19.17, *p* < 0.001), with a Nagelkerke R^2^ = 0.10. Political self-identification (β = 0.612, *p* = 0.001) was the only variable significantly associated with the outcome in the model of the perceived importance of CSE. Specifically, more liberal teachers were more likely to report higher perceived importance.

In contrast, years of teaching experience (β = −0.162, *p* = 0.065), educational level (β = −0.058, *p* = 0.734) and frequency of religious attendance (β = −0.225, *p* = 0.125) were not statistically significant predictors (Table 9).

## 5. Discussion

The present study examined parents’ and teachers’ perceptions of CSE in preschool and primary education, focusing on its perceived importance, appropriate timing and content, implementation barriers, and the influence of socio-political and demographic factors. Overall, both groups expressed strong support for CSE, reinforcing previous findings that highlight its perceived value for children’s development and wellbeing (Zhuravleva & Helmer, 2024).

The findings of this study converge around a central pattern: although both parents and teachers support CSE at a general level, this support becomes conditional and selective when specific content areas—particularly those related to sexuality and sexual behaviour—are considered. This suggests that support for CSE is not unconditional but shaped by underlying moral and socio-cultural evaluations.

Participants primarily emphasised the protective role of sexuality education, particularly in preventing sexual abuse and helping children distinguish between appropriate and inappropriate touch (Özel et al., 2023). Similarly, concerns about misinformation reflect broader findings indicating that, in the absence of structured education, children may rely on unreliable sources (Zhuravleva & Helmer, 2024). These findings suggest that both parents and teachers tend to conceptualise CSE mainly as a protective and preventive mechanism, rather than as a fully comprehensive and rights-based educational framework.

At the same time, notable differences emerged between parents and teachers. Parents expressed greater concern regarding age appropriateness, potential loss of innocence, and fears of risky experimentation. These concerns may be interpreted through the lens of protective morality, whereby attitudes are shaped by the desire to shield children from perceived premature exposure to sexuality-related content (P. Martin et al., 2020). In contrast, teachers appeared less likely to endorse such concerns, which may reflect their professional experience and familiarity with children’s everyday questions and developmental needs.

This conditional pattern is also reflected in perceptions of the appropriate age for introducing CSE. Parents tended to favour later introduction, primarily during primary school, whereas teachers supported earlier implementation, including in preschool. This divergence may reflect differing role expectations, with parents adopting a more protective stance and teachers aligning more closely with developmental and pedagogical frameworks. This pattern is consistent with international research indicating that educators are generally more supportive of early, developmentally appropriate CSE than parents (Depauli & Plaute, 2018; Plaza-del-Pino et al., 2021).

Both groups demonstrated a developmental approach to CSE content, prioritising relational and protective topics in early childhood and gradually endorsing more complex themes at later stages. However, resistance was concentrated on specific topics, particularly those directly related to sexuality and sexual behaviour. This pattern—high overall support combined with selective resistance—constitutes a key finding of the study. Rather than indicating inconsistency, it suggests that perceptions toward CSE are structured through differentiated moral evaluations. Topics perceived as protective (e.g., safety, relationships) are widely accepted, whereas topics associated with sexuality, sexual behaviour and reproduction are more likely to be contested. This aligns with the concept of moral framing, whereby concerns related to protection, childhood innocence, and social norms shape perceptions of age-appropriate content (Cacciatore et al., 2020; Haidt, 2012; Robinson & Davies, 2017).

Moreover, this pattern of conditional support reflects broader trends observed across the European region, where the implementation and public acceptance of CSE remain uneven and heterogeneous across countries and socio-cultural contexts (Ketting et al., 2021). Although CSE is widely recognised as an important educational framework, resistance tends to emerge selectively and is often concentrated around specific themes, particularly those related to sexuality, sexual behaviour, gender identity, and sexual orientation (Sell et al., 2023). In contrast, topics associated with child protection, bodily safety, and basic health knowledge generally receive broader public acceptance. Previous research has also shown that debates surrounding CSE in early childhood are frequently shaped by concerns about childhood innocence, fears of premature sexualisation, and questions regarding age appropriateness (K. A. Martin & Bobier, 2017). These findings align closely with the present study, in which participants expressed broad support for protective and relational dimensions of CSE while demonstrating greater hesitation toward sexuality-related content. Similar patterns of broad support accompanied by reservations regarding specific sexuality-related topics and early childhood implementation have also been documented in previous stakeholder-focused research conducted in the Greek educational context (Katsarou & Pliogou, 2026).

Within the Greek context specifically, previous research similarly highlights the persistence of dominant discourses centred on childhood innocence, age appropriateness, and strong parental control over sexuality-related knowledge in early childhood (Grigoropoulos, 2025). These discourses may function as normative frameworks that shape public perceptions and limit the acceptance of more comprehensive aspects of CSE. This pattern was also reflected in the present findings, where participants expressed broad support for protective and relational dimensions of CSE, while demonstrating greater hesitation toward topics directly related to sexuality, sexual behaviour, and reproduction. In particular, parents were more likely than teachers to express concerns regarding age appropriateness, premature exposure, and the potential loss of childhood innocence, suggesting that support for CSE may be shaped by protective understandings of childhood.

At the same time, resistance appeared to be more evident in relation to sexuality-related topics, which remain politically and socially contested, especially in more conservative socio-cultural contexts (Datta, 2018; Kuhar & Zobec, 2017; Michielsen & Ivanova, 2022). These tensions highlight the ongoing challenges in reconciling rights-based approaches to CSE with prevailing cultural norms and underline the importance of considering the wider socio-political environment in the interpretation of perceptions toward sexuality education.

This conditional acceptance is further reflected in the barriers identified by participants. Teachers were more likely than parents to identify lack of training, lack of confidence, parental resistance, and broader social conservatism as key obstacles. Similar concerns regarding insufficient teacher preparation, institutional support, and sociocultural resistance have also been documented in previous research examining stakeholders’ perspectives on CSE implementation in Greece (Katsarou & Pliogou, 2026). This pattern suggests that educators may be more aware of structural and institutional constraints, as well as of the practical challenges involved in implementing CSE in classroom settings. At the same time, these findings may reflect broader elements of cultural conservatism within the Greek context, as reflected in this regional sample, where sexuality-related topics remain sensitive and are often negotiated between traditional values and contemporary educational approaches.

The evaluation of educational policy also reflects this tension. Both parents and teachers perceived current policy as largely inadequate, pointing to a gap between policy intentions and classroom practice (Depauli & Plaute, 2018). Parents’ more negative evaluations, combined with their perception that Greek society is not yet ready to accept CSE, may indicate lower levels of trust in the broader implementation context. In contrast, teachers’ relatively more moderate views may suggest a degree of professional confidence in the potential for implementation, despite recognising existing limitations.

While both groups recognised the importance of specialised knowledge, parents were more likely to emphasise their own role in decision-making, whereas teachers more frequently highlighted the role of educators and the Ministry of Education. This tension between family and institutional authority has already been documented (Ketting & Ivanova, 2018) and may reflect differing perceptions of responsibility and control over children’s socialisation.

Beyond the importance of specialised training, some participants also emphasised personal characteristics of educators delivering CSE, such as friendliness, approachability, and interpersonal communication skills. These findings further support previous stakeholder-based research conducted in Greece (Katsarou & Pliogou, 2026), suggesting that CSE providers are expected to combine scientific knowledge with interpersonal and emotional competencies that facilitate safe and trusting communication with children.

Although only a very small minority of participants expressed gender-based preferences regarding who should provide CSE—for example, suggesting that male educators may be more appropriate for boys and female educators for girls—it is notable that such views were reported not only by parents but also by some teachers. This may reflect the persistence of gendered assumptions surrounding communication about sexuality and the perceived appropriateness of discussing sexuality-related topics with children, even within educational contexts. From a gender perspective, such assumptions may be understood as socially constructed expectations linked to broader cultural expectations regarding gender, caregiving, and sexuality (Butler, 1990; Connell, 2009).

Finally, ideological orientation appeared to show the most consistent association with the perceived importance of CSE across both samples, with more liberal participants tending to report more positive views, in line with previous research (Kantor & Levitz, 2017). This finding supports the interpretation that the perceived importance of CSE is embedded within broader systems of values and moral frameworks.

In the parent sample, educational level also appeared to be associated with the perceived importance of CSE; however, consistent with previous literature, this relationship appears less stable and may operate in interaction with ideological and cultural factors rather than as an independent predictor (Kee-Jiar & Shih-Hui, 2020).

Religious attendance was negatively associated with the perceived importance of CSE at the bivariate level in both groups and remained statistically significant only among parents in the multivariate model, although this finding should be interpreted with caution given the limitations of the ordinal regression model. This finding aligns with research suggesting that religious attendance does not uniformly predict opposition to CSE but may strengthen more conservative value orientations, particularly when combined with ideological factors (Hendriks et al., 2024; Hurst et al., 2023; Grigoropoulos, 2025).

Although the regression models were statistically significant, their explanatory power was modest (Nagelkerke R^2^ = 0.18 for parents and 0.096 for teachers), indicating that a substantial proportion of variance in attitudes toward CSE remains unexplained. This suggests that additional cultural, contextual, and psychological factors not included in the present models may contribute to shaping these perceptions.

Finally, the potential influence of social desirability bias should be considered, given the sensitive nature of sexuality-related topics. Participants may have expressed high levels of general support while adopting more restrictive positions when responding to specific or sensitive content areas.

Overall, the findings indicate that attitudes toward CSE are shaped by interconnected value systems in which ideological orientations, moral evaluations, and socio-cultural norms interact. The coexistence of general support with selective resistance highlights that the acceptance of CSE is conditional and context-dependent. While ideological orientation emerged as a key predictor of the perceived importance of CSE, the relatively low explanatory power of the models suggests that this specific dimension cannot be fully explained by socio-demographic variables alone. Taken together with the broader pattern of findings, this points to the role of wider cultural meanings attached to childhood, morality, and sexuality in shaping perceptions of CSE. At the same time, the generally small effect sizes observed across many group comparisons suggest substantial overall convergence between parents’ and teachers’ attitudes toward CSE, despite selective differences regarding specific themes and implementation issues.

## 6. Limitations

This study has several limitations. First, the study was conducted in the Epirus region of Greece. While the findings cannot be generalised to the entire country, the region provides an informative case study of early childhood education within a national system characterised by centralised curricula and shared teacher training structures. Second, the reliance on self-reported data may have introduced social desirability bias. Third, the use of an online questionnaire may have influenced participation, particularly among parents with limited digital access. In addition, mothers were overrepresented in the parent sample, reflecting their greater involvement in school-related activities and research participation. Consequently, the findings may be more reflective of mothers’ perspectives than those of fathers and should be interpreted with this consideration in mind. Finally, it cannot be confirmed whether all school principals distributed the questionnaire to parents, which may have affected sample representativeness. In addition, the selective reporting of items common to both the parents’ and teachers’ questionnaires, drawn from broader instruments, limits the possibility of examining latent constructs across the full set of measures. As a result, the dimensional structure of the constructs could not be assessed, and future research may benefit from the use of multi-item validated scales to enable more robust psychometric analyses.

## 7. Implications for Policy and Practice

A key implication concerns the need for systematic and sustained teacher training. Both groups identified specialised training as essential, and teachers themselves highlighted lack of preparation and confidence as major barriers. Therefore, integrating CSE into both pre-service and in-service teacher education programs is critical for effective implementation.

Second, clear and developmentally structured curricula are needed to address differences between parents and teachers regarding timing and content. Aligning national curricula with international guidelines proposed by UNESCO (2018), could help address parental concerns by emphasising the progressive and age-sensitive nature of CSE.

Third, the findings highlight the need for stronger communication and collaboration between schools and families. Providing parents with clear information about the aims and content of CSE may reduce resistance and enhance trust.

Furthermore, the perceived inadequacy of current policy underscores the need for more coherent implementation strategies, including teaching materials, institutional support, and context-sensitive approaches.

Finally, the influence of ideological and religious factors suggests that policy approaches should be sensitive to the broader socio-cultural context.

## 8. Conclusions

The present study demonstrates strong overall support for CSE, particularly when framed as a means of protecting children and promoting informed understanding. At the same time, the findings reveal an important underlying tension: while CSE is broadly accepted at a general level, more selective and restrictive perceptions emerge in relation to specific content areas, particularly those related to sexuality and reproduction.

The results suggest that the main challenge is not whether CSE should be introduced, but how it can be implemented in ways that are developmentally appropriate, pedagogically sound, and socially supported. Addressing this conditional acceptance requires careful consideration of the moral and socio-cultural frameworks that shape stakeholders’ perceptions.

Strengthening teacher training, enhancing policy coherence, and fostering collaboration between schools and families emerge as critical priorities for the effective implementation of CSE in early educational settings.

## Figures and Tables

**Table 1:** Demographics.

Variable	Category	Parents *n* (%) *	Teachers *n* (%) *
Gender	Female	261 (80.6)	165 (78.2)
	Male	63 (19.4)	46 (21.8)
Age	20–29	7 (2.1)	2 (0.9)
	30–39	112 (34.4)	27 (12.7)
	40–49	179 (54.9)	71 (33.5)
	50+	28 (8.6)	112 (52.8)
Education	Basic	81 (24.7)	-
	University degree	147 (45.1)	80 (37.7)
	Second degree	-	12 (5.7)
	Postgraduate	88 (27.0)	109 (51.4)
	PhD	10 (3.1)	11 (5.2)
Residence	Urban	231 (71.5)	-
	Semi-urban	33 (10.2)	-
	Rural	59 (18.3)	-
Income	≤10,050 €	38 (11.8)	-
	10,051–20,000 €	130 (40.2)	-
	20,001–30,000 €	89 (27.6)	-
	>30,001 €	66 (20.4)	-
Teaching experience	0–5 years	-	15 (7.1)
	6–15 years	-	31 (14.7)
	16–25 years	-	90 (42.6)
	25+ years	-	75 (35.5)
School type	Kindergarten	-	111 (52.9)
	Primary school	-	99 (47.1)
Political self-orientation	Centre	172 (52.8)	94 (44.3)
	Somewhat liberal	89 (27.3)	82 (38.7)
	Very liberal	33 (10.1)	22 (10.4)
	Somewhat conservative	18 (5.5)	7 (3.3)
Religious attendance	Never	30 (9.2)	12 (5.7)
	Once a year	37 (11.3)	34 (16.1)
	Few times per year	203 (62.3)	125 (59.2)
	Few times per month	36 (11.0)	21 (10.0)
	Weekly	14 (4.3)	13 (6.2)
	>Weekly	-	4 (1.9)

* Percentages may not sum to 100 due to rounding and missing values.

**Table 2:** Reasons for providing CSE in schools (multiple responses).

Reasons	Parents *n* (%) * N = 311	Teachers *n* (%) * N = 212
Understanding the difference between appropriate and inappropriate touch	269 (86.5)	187 (88.2)
Reducing sexual abuse	267 (85.9)	189 (89.2)
Preventing misinformation from unreliable sources	255 (82.0)	176 (83.0)
Reducing sexually transmitted infections	226 (72.7)	125 (59.0)
Reducing teenage pregnancies	225 (72.3)	139 (65.6)
It is necessary for children to know	223 (71.7)	141 (66.5)
Preventing children from asking uncomfortable questions	53 (17.0)	40 (18.9)

* Multiple responses were allowed; therefore, percentages do not sum to 100%.

**Table 3:** Reasons for not providing CSE in schools (multiple responses).

Reasons	Parents *n* (%) * *N* = 80	Teachers *n* (%) * *N* = 101
Children are too young	40 (50.0)	30 (29.7)
Children lose their innocence	36 (45.0)	24 (23.8)
Leads to risky experimentation	35 (43.8)	26 (25.7)
We never received sexuality education, therefore it is not necessary	6 (7.5)	—
Lack of teacher training	3 (3.8)	—
Parents know better how and when to inform their children	1 (1.3)	—
The people providing CSE may not be appropriate	—	4 (4.0)
It may lead to conflicts with parents	—	3 (3.0)
Teachers’ stereotypes and prejudices may negatively influence students	—	2 (2.0)

* Multiple responses were allowed; therefore, percentages do not sum to 100%.

**Table 4:** Importance of key concepts.

Key Concept	Parents *n* (%) *	Teachers *n* (%) *
Relationships	622 (13.3)	413 (13.6)
Values, Rights, Culture and Sexuality	602 (12.9)	428 (14.1)
Understanding Gender	520 (11.1)	366 (12.1)
Violence and Staying Safe	499 (10.7)	307 (10.1)
Skills for Health and Well-being	648 (13.9)	474 (15.6)
The Human Body and Development	816 (17.5)	403 (13.3)
Sexuality and Sexual Behaviour	352 (7.5)	258 (8.5)
Sexual and Reproductive Health	606 (13.0)	382 (12.6)
Total Responses	4665 (100)	3031 (100)

* Multiple responses were allowed; therefore, percentages do not sum to 100%.

**Table 5:** Age appropriateness of key concepts.

Key Concept	Age	Parents (%) *	Teachers (%) *
Relationships	5–6 years	5.74	5.77
	7–9 years	4.28	4.18
	9–12 years	4.10	4.85
	Not at all	0.96	0.73
Values, Rights, Culture and Sexuality	5–6 years	1.13	1.56
	7–9 years	3.24	2.63
	9–12 years	5.70	5.76
	Not at all	0.66	0.83
Understanding Gender	5–6 years	2.34	2.95
	7–9 years	3.64	2.95
	9–12 years	4.53	4.74
	Not at all	0.84	0.70
Violence and Staying Safe	5–6 years	3.05	3.76
	7–9 years	3.89	3.68
	9–12 years	4.50	4.26
	Not at all	0.50	0.65
Skills for Health and Well-being	5–6 years	3.06	4.19
	7–9 years	5.91	6.08
	9–12 years	8.53	8.11
	Not at all	1.17	1.25
The Human Body and Development	5–6 years	2.30	2.95
	7–9 years	4.03	3.43
	9–12 years	8.00	7.40
	Not at all	0.93	1.09
Sexuality and Sexual Behaviour	5–6 years	0.23	0.39
	7–9 years	1.15	0.95
	9–12 years	4.38	3.99
	Not at all	1.11	1.12
Sexual and Reproductive Health	5–6 years	0.21	0.37
	7–9 years	1.21	0.75
	9–12 years	7.23	6.33
	Not at all	1.34	1.70

* Percentages are based on the total number of responses (*N* = 8946 parents, *N* = 6415 teachers), as participants were allowed to select multiple options.

**Table 6:** Barriers to implementation of CSE.

Barrier	Parents *n* (%) * *N* = 321	Teachers *n* (%) * *N* = 212
Lack of teacher training	229 (71.3)	183 (86.3)
Parents’ fear	180 (56.1)	158 (74.5)
Lack of educational material	159 (49.5)	101 (47.6)
Conservatism of Greek society	156 (48.6)	128 (60.4)
Parents do not want CSE being taught	145 (45.2)	121 (57.1)
Teachers feel uncomfortable teaching CSE	104 (32.4)	138 (65.1)
Objections from institutions (e.g., Church)	97 (30.2)	80 (37.7)
Not included in mandatory SL	86 (26.8)	42 (19.8)
Lack of suitable subject area	79 (24.6)	44 (20.8)
Lack of political will	75 (23.4)	47 (22.2)
Children lose innocence	42 (13.1)	19 (9.0)

* Multiple responses were allowed; therefore, percentages do not sum to 100%.

**Table 7:** Political self-identification and perceived importance of CSE.

Political Self-Identification	Parents (M/SD, *N*)	Teachers (M/SD, *N*)
Very liberal	4.42/1.00 (*N* = 33)	4.59/0.50 (*N* = 22)
Somewhat liberal	4.29/0.77 (*N* = 89)	4.15/0.85 (*N* = 82)
Centrist	3.76/1.05 (*N* = 171)	4.01/0.78 (*N* = 94)
Somewhat conservative	3.47/1.23 (*N* = 17)	4.00/0.57 (*N* = 7)
Kruskal–Wallis (H, *p*)	H(3) = 32.06, *p* < 0.001 ***	H(3) = 11.46, *p* = 0.009 **

*p* < 0.01 **, *p* < 0.001 ***.

**Table 8:** Ordinal logistic regression analysis (parents). (Dependent variable: perceived importance of CSE.).

Variable	B	SE	*p*
Educational level	0.504	0.150	<0.001 ***
Household income	−0.220	0.122	0.071
Place of residence	−0.671	0.440	0.127
Political self-identification	0.679	0.164	<0.001 ***
Religious attendance	−0.360	0.143	0.012

*p* < 0.001 ***.

**Table 9:** Ordinal logistic regression analysis (teachers). (Dependent variable: perceived importance of CSE.).

Variable	B	SE	*p*
Years of teaching experience	−0.162	0.088	0.065
Educational level	−0.058	0.170	0.734
Political self-identification	0.612	0.193	0.001 ***
Religious attendance	−0.225	0.147	0.125

*p* < 0.001 ***.

## Data Availability

Data are unavailable due to privacy restrictions.

## Abbreviations

The following abbreviations are used in this manuscript:

CSE	Comprehensive Sexuality Education
LGBTQ	Lesbian, Gay, Bisexual, Transgender, Queer, Intersex, other identities
SL	Skills Labs
